# Interventions to reduce work-related musculoskeletal disorders among healthcare staff in nursing homes; An integrative literature review

**DOI:** 10.1016/j.ijnsa.2021.100033

**Published:** 2021-05-31

**Authors:** Enobong Gideon Asuquo, Sylvia Murphy Tighe, Carmel Bradshaw

**Affiliations:** a Catherine McAuley House Nursing Home, Old Dominic street, Limerick, Ireland; b Dept. of Nursing and Midwifery, University of Limerick, Health Research Institute (HRI) affiliated, Limerick, Ireland; c Dept. of Nursing and Midwifery, University of Limerick, Health Research Institute (HRI) affiliated, PG Dip Education (Midwifery), Limerick, Ireland

**Keywords:** Work-related musculoskeletal disorder, Healthcare workers, Nursing homes, Long term residential home, Moving and handling older people, Injury prevention

## Abstract

**Background:**

: The incidence of work**-**related musculoskeletal disorders have been consistently high in nursing sectors and are a significant cost to the health service due to absenteeism. Work**-**related musculoskeletal disorders are more common among healthcare workers in nursing homes due to the high dependency needs of older persons which often include need for help with self-care and mobility. Therefore, staff are exposed to potential injury associated with moving and handling patients. Work**-**related musculoskeletal disorders significantly impact on the quality of life of affected individuals, can cause economic hardship and affect service provision.

**Aim:**

: To identify, review, and discuss interventions that may be effective in reducing the prevalence and consequences of work**-**related musculoskeletal disorders in nursing homes.

**Design:**

: An integrative literature review.

**Method:**

: A systematic search of Embase, Science direct, Web of science and EBSCO Host was conducted and an ancestry search of the references of the reviewed articles were also reviewed. Peer reviewed primary research published between 2000 and 2020 were selected. The quality of these studies was appraised with Crowe Quality Appraisal Tool and reported using the Preferred Reporting Items for Systematic Reviews and Meta-Analyses guidelines. The components of the interventions were categorised using Burnard (2011) framework for content analysis.

**Results:**

: Fifteen studies met the criteria for inclusion in the review. Interventions reported in the literature were in four categories; (i) Specialised equipment (ii) Staff training (iii) Policies and procedures to reduce work-related musculoskeletal disorders and (iv) Support and follow up. A ceiling lift is the intervention of choice to reduce work**-**related musculoskeletal disorders, as it reduces the stress associated with pushing and pulling. Risk assessment is vital to determine the individual needs of clients for safe handling. Train-the-trainer roles could be used to implement training interventions where resources limit the employment of a designated lead to facilitate injury prevention. A multifaceted approach to prevent and reduce work**-**related musculoskeletal disorders is recommended. Further research is required to ascertain the effectiveness of Cognitive Behavioural Therapy on injury reduction in nursing homes.

**Conclusion:**

: These findings have the potential to inform the development and adherence to injury prevention policies and regulations by healthcare managers and staff which could reduce injuries. Identifying the appropriate interventions to prevent and reduce work**-**related musculoskeletal disorders is significant for staff wellbeing, has economic implications in terms of reduced work**-**related musculoskeletal disorder absenteeism and ultimately will positively impact on the care of mobility impaired clients.

**What is already known about this topic?**

• The prevalence of work-related musculoskeletal disorder is higher in nursing homes than in other nursing sectors.
• Injury and absenteeism negatively impact the quality of patient care, affects the quality of life of the healthcare worker which can lead to economic hardship for the individual and compensation claims are a burden on the health services.
• Many studies have examined how musculoskeletal disorders could be alleviated in nursing homes, but the prevalence of injury is still high, therefore, evidence-based guidance on preventive measures are required.

**What this paper adds:**

• This paper synthesises the evidence (2000–2020) on means of preventing and reducing work-related musculoskeletal injury in nursing homes.
• The use of ceiling lifts for clients appear to be more effective in reducing injury than the use of floor lifts.
• A multidimensional approach which includes participatory staff training and regular follow up is a key factor in reducing injury in nursing homes.
• A designated person within an organization, championing preventive practices in relation to work-related musculoskeletal injury could play a significant role in reducing injuries.

## 1 Introduction

Work-related musculoskeletal disorders are prevalent in many occupations worldwide (Aghilinejad et al., 2012; Davis and Kotowski 2015). Work**-**related musculoskeletal disorders is a broad term used to describe harmful conditions caused by overuse of some parts of the muscles, tendons, nerves, ligaments, joints, and supporting blood vessels, as an outcome of a work-related activity (Russell et al., 2016; Wang et al., 2017). Work**-**related musculoskeletal disorders include low back pain, shoulder pain, repetitive strain injuries, joint pain (knees, wrists and other joints) which hinders body movement (Punnett and Wegman 2004; Davis and Kotowski 2015). The term work**-**related musculoskeletal disorders is synonymous with musculoskeletal injuries and musculoskeletal disorders among others (Da Costa and Vieira 2010; Lee et al., 2015). While work**-**related musculoskeletal disorders are common among workers, nurses are more likely to develop a work**-**related musculoskeletal disorder than other professionals as their job is often physically demanding (Davis and Kotowski 2015).

Within the nursing sector, the highest rate of work**-**related musculoskeletal disorders globally have persistently been reported among nurses employed in long-term care settings. (Kromark et al., 2009; Gold et al., 2017). A European Nurses Exit (NEXT) study of seven countries revealed that 63.5% of nurses in the nursing home setting left the profession as a result of suffering from work**-**related musculoskeletal disorders (Simon et al., 2008). A German study by Kromark et al. (2009) found that health care staff in the nursing home settings are seven times more likely to suffer lower back pain compared with those delivering home care.

Smith et al. (2003) also reported a higher prevalence of work**-**related musculoskeletal disorders among healthcare staff in nursing homes in North Korea than in other occupational sectors where manual handling, changing patients’ clothes and working as a healthcare assistant are identified as risk factors. Other risk factors for work**-**related musculoskeletal disorders identified in similar studies include rapid repetitive work movements, non-aligned body postures and caring for non-cooperative clients (Collins et al., 2006; Russell et al., 2016). Furthermore, studies have shown that the higher the dependency level of clients, (which is commoner in nursing homes) the higher the need for assistance with care, which increases the risk of work**-**related musculoskeletal disorders among nursing home staff compared with other nursing specialities (Kurowski et al., 2017).

Work**-**related musculoskeletal disorders are of great concern for workers and employers as they carry a high cost of absenteeism, medical treatment cost, workplace injury compensation, and permanent musculoskeletal limitations. (Tompa et al., 2016; O'Brien et al., 2019). These factors affect the quality and availability of care for older people. The World Health Organisation (2018) estimates the population aged 60 and above to increase from 900 million in 2015 to two billion in 2050. These projections will require an increased number of healthy staff to provide healthcare services to older people where impaired mobility is often a factor (Ellapen and Narsigan 2014).

There is recognition that there has been underreporting and poor documentation of work**-**related musculoskeletal disorders due to self-reporting, recall bias, and the difficulties with establishing cause and effects. Despite this, the prevalence of reported work**-**related musculoskeletal disorders is still high (Carugno et al., 2012; Harcombe et al., 2014). In 2018/2019, the United Kingdom lost an estimated 6.9 million workdays due to work**-**related musculoskeletal disorders, accounting for 29% of all days lost (HSE-UK 2019). In Ireland, the Health and Safety Authority (HSA-Ireland 2020) reported that the health sector recorded work-related injuries of 21.4%, the highest annual rate of work**-**related musculoskeletal disorders recorded to date in Ireland. Furthermore, an Irish study revealed that the highest rate of work**-**related musculoskeletal disorders related sick leave is among nursing staff (Cunningham et al., 2006). This high prevalence, thus, necessitates the understanding of preventive measures for work**-**related musculoskeletal disorders to improve health among healthcare workers and reduce the costs of absenteeism to the health services, whilst continuing to provide quality care to those that are in need (Andersen and Mikkelsen 2008).

Healthcare assistants, sometimes referred to as nursing assistants, nursing aides and/or carers are categorised with nurses as the core healthcare staff in nursing homes (Davis and Kotowski 2015; O'Brien et al., 2019) and are equally at an increased risk of work**-**related musculoskeletal disorders because of the physicality of their work. Thus, interventions to prevent work**-**related musculoskeletal disorders should target both Health Care Assistants and nurses (Kurowski et al., 2012).

There is some evidence available on interventions that could prevent or reduce the severity of work**-**related musculoskeletal disorders (Miller et al.,2005; Tompa et al., 2016; Gold et al., 2017).

However, a search of the literature failed to find evidence of a review of research which focused on the specific needs of staff working in nursing homes in relation to interventions to prevent or reduce work**-**related musculoskeletal disorders. Therefore, this review aims to identify, review, and discuss research findings specific to interventions to prevent or reduce work**-**related musculoskeletal disorders among healthcare staff (Nurses and Healthcare assistants) working in nursing homes. This integrative review seeks to determine what interventions could reduce work-related musculoskeletal injuries among health care staff in nursing homes, which could improve work**-**related musculoskeletal disorders related morbidity, reduce absenteeism, inform policies and practice and thus improve the quality of care to older persons.

## 2 Methods

### 2.1 Design

An integrative literature review method was employed as an appropriate review method as it allows for the inclusion of studies with diverse methodologies which broadens the evidence for the review (Whittemore and Knafl 2005). The PICO format (Population, Intervention, Comparison, Outcome) was utilised to develop the research question (Santos et al., 2007), see table 1 which also identifies the inclusion and exclusion criteria for the review. Relevant studies from the published literature were identified, synthesised and analysed culminating in a comprehensive description of interventions to reduce work**-**related musculoskeletal disorders.

**Table 1:** PICO and Inclusion and exclusion criteria.

PICO Terms	Inclusion	Exclusion
Population	Nurses and Healthcare Assistants whose works include physical movement of clients.	Other health professionals in nursing homes not directly involved in physical care of the clients. Example household, catering and administration staff.
Setting	Long-term facility for the aged; Nursing homes, Geriatric rehabilitation centres, assisted living facility.	Short-term facility for the aged. Example geriatric units of hospitals and day care centres for the older persons. Studies conducted outside the healthcare setting, for example in the laboratory are excluded.
Intervention	All actions, interventions targeting the reduction of work**-**related musculoskeletal disorders and its related cost among nurses and healthcare assistants. Both single and multicomponent interventions included.	Actions, interventions that enhance staff wellbeing but do not specifically address work**-**related musculoskeletal disorders.
Types of study	Primary studies, Peer reviewed articles published in English language. Studies of all designs are included.	Studies published in non-peer reviewed journals. Government agencies and departmental publications, PhD thesis.
Date	Published between 2000 and 2020 (20 years).	All studies published prior to 2000 and after 2020

**Fig. 1:**
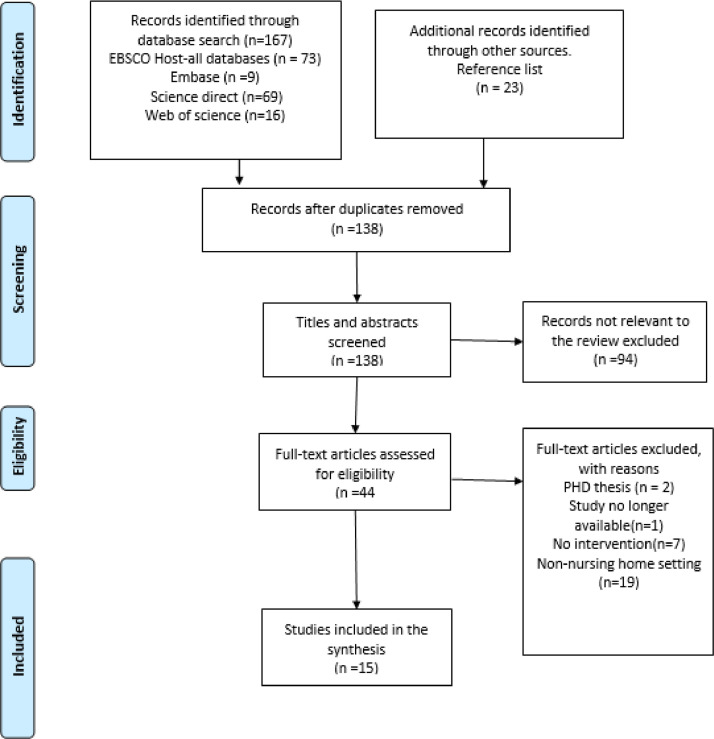
PRISMA FLOW DIAGRAM. Source: Moher et al. (2009).

To ensure transparency in the literature search, the PRISMA (Preferred Reporting Items for Systematic Reviews and Meta-Analyses) flow diagram (Fig. 1) was used in reporting the search process. The Crowe Quality Appraisal Tool was used to appraise the quality of the studies.

Given the quantitative nature of most of the studies in this review, a Content Analysis approach was considered suitable in identifying the frequency of occurrence of specific interventions (Krippendorff 2018). Burnard's (2011) framework for content analysis was applied which necessitated reading and re-reading the studies, extracting the interventions into a format referred to as codes that were subsequently grouped into patterns called categories (Details in Supplemental material 4).

### 2.2 Search strategy

The literature search covered a 20-year period (2000–2020). In the initial scoping search, seven relevant studies were identified between 2000 and 2010 and this prompted the choice of the year 2000 as the search start date in order to include such studies in the review.

The aim of a search spanning 20 years was to enhance an extensive literature search (Benavides et al., 2010) and increase the chances of identifying all studies on interventions that reduce work**-**related musculoskeletal disorders. A systematic search of EBSCO Host, Science direct, Web of science and Embase was completed. An ancestry search of the reference lists of included studies was also carried out to ensure an extensive search as advised by Whittemore and Knafl (2005). Boolean operators (AND, OR) were used in Embase, Web of Science, and Science direct search while both Boolean operators and truncation (for example nurs* for nurse, nurses, nursing) were used together for the search in Ebsco Host. The different search strategies were applied as it suited each database search platform. (Bettany-Saltikov 2016). Table 2 displays the details of search terms used in different databases. The initial search was done by the first author and confirmed by the second and third author (initials to be inserted). Data were extracted by reading each study and recording relevant information into a data extraction form (Table 3).

**Table 2:** Details of database searches.

Ebsco host all data bases		Embase	Science Direct	Web of Science
Number of searches	Search terms	Combined search terms	Combined search terms	Combined search terms
S1	musculoskeletal disorder* OR musculoskeletal injur* OR musculoskeletal discomfort*	('musculoskeletal injuries' OR (musculoskeletal AND ('injuries'/exp OR injuries))) AND ('healthcare workers' OR (('healthcare'/exp OR healthcare) AND workers)) AND ('prevention'/exp OR prevention) AND ('nursing homes'/exp OR 'nursing homes' OR (('nursing'/exp OR nursing) AND homes))	work related musculoskeletal injuries AND nurses AND healthcare assistants AND nursing homes AND prevention	**ALL FIELDS:** (musculoskeletal injuries) *AND***ALL FIELDS:** (nursing staff) *AND***ALL FIELDS:** (nursing homes) *AND***ALL FIELDS:** (prevention)
**S2**	nurs* OR nurs* aid* OR healthcare assist*			
**S3**	nursing home OR long-term care facility OR old people's home			
**S4**	prevent* OR reduc* OR decreas*			
**S5**	# S1 AND # S2 AND # S3 AND # S4

**Table 3:** Data extraction table.

Author(s), Year, Country and CCAT Score	Study purpose	Study design	Sample	Setting	Data collection Method	Interventions	Outcomes
Peterson et al. (2004)**United States of America.** 78%	To develop an ergonomics training program for selected Health Care Assistants at a state-run veterans' home to decrease musculoskeletal disorders.	Experimental	Health Care Assistants. *n* = 35	Nursing home	Questionnaire, Videotaping	Assessing the work environment, developing and application of a training program by Healthcare Assistants.	There was no significant reduction in pain, discomfort or general health status between the three study groups.
Collins et al. (2004). **United States of America.** 83%	To conduct an intervention trial of a ‘‘best practices’’ musculoskeletal injury prevention program designed to safely lift physically dependent nursing home residents. .	Pre and post intervention evaluation	Nursing staff *n* = 1728	6 Nursing homes	Injury records, compensation claims, logbook.	-Mechanical lifts and repositioning aids. - A lift policy. -Employee training.	There was significant decrease in client handling injury and lost workdays at 95% confidence interval -$55,000 annual compensation savings, and lost workdays.
Kozak et al. (2017). **Germany.** 90%	To use measurement in evaluating the effectiveness of a training program on the reduction of stressful trunk postures among healthcare professionals working with older peoples.	Experimental	Nurses and Healthcare Assistants. *n* = 19	6 nursing homes	Computer-Assisted Recording and Long-Term Analysis of Musculoskeletal Loads measurement system	-Instruction on body postures during nursing care -physical exercises -reorganization of the work environment	- Staff maintained proper ergonomic measures with 29% reduction in time spent in sagittal inclination. - Reduction in stressful trunk postures from 2.5% to 1.0%.
Brophy et al. (2001). **United States of America***.* 73%	To reduce musculoskeletal injuries in employees and lower the financial costs associated with them. The goal of the ergonomics intervention implemented in this study was to change not only the workplace environment by introducing lifting equipment, but also the workplace culture.	Experimental	All Healthcare Assistants in a 525- bed facility.	Nursing home	Injury and compensation records.	-Formation of a resident transfer evaluation team and accident appraisal committee -Compulsory ergonomics training for new Healthcare Assistants -Regular maintenance checks for lifting equipment -Direct access to the management and budget process.	-Reports of lower back pain reduced from 15.7 to 11per 100 Healthcare Assistants. -Total number of lost workdays reduced from 1476 to 625 annually. -The annual cost related to lower back pain reduced from $201,100 to $91,800.
Alamgir et al. (2008). **Canada.** 88%	To evaluate the effectiveness and cost benefit of overhead lifts in reducing the risk of musculoskeletal injury among healthcare workers.	Pre/post intervention analysis	All Nurses and Healthcare Assistants in a 455 -bed facility	Nursing home, extended care centres	Injury and compensation claims records	Installation of ceiling lifts	Significant decline in injury rates and compensation cost savings of Can $ 11, 657.21.
Miller et al. (2006). **Canada.** 80%	To assess the effectiveness of portable ceiling lifts in a new multi-level care facility on risk of patient handling injuries where the ratio of ceiling lifts to resident beds is one to six.	Experimental	Nurses and Healthcare Assistants. *n* = 74	Nursing homes	Pre and post intervention questionnaire	The installation of portable ceiling lifts	−75% of staff always preferred to use the ceiling lifts for patient transfer. −241% increase in Compensation claims in the comparison facility.
Gold et al. (2017). **United States of America.** 83%	To examine whether Lower Back Pain declined post intervention and whether it was associated with relevant self-reported occupational exposures or personal health factors.	Pre/post intervention review.	Nurses and Healthcare Assistants *n* = 1154	Nursing home	Questionnaire	-The provision of mechanical lifts. -Establish rules for equipment and sling maintenance. -Training of staff.	-Decline to 37% and 22.4% of lower back pain at 2 and 6 years. −95% confidence interval of protective effects from frequent lift device use and constant exercise activities.
Kurowski et al. (2017) **United States of America.** 93%	To reduce the incidence and cost of work**-**related musculoskeletal disorders	Post intervention study	Nurses and Healthcare Assistants. *n* = 11,603	Nursing homes	Workers’ compensation data	-Purchase of mechanical lifting devices. -Workers training. -Availability of usage/maintenance procedures.	−11% and 14% reduction in staff compensation claims in the first and second year post intervention.
Kurowski et al. (2012) **United States of America.** 75%	To examine the effect of a safe resident handling intervention on the ergonomic exposures of nursing assistants.	Pre and post intervention study	Healthcare Assistants and Nurses. (99,000 observations)	Nursing homes	Observation	-Mechanical devices for patient handling. -Job training for staff. -Equipment use and maintenance protocols.	-Increased likelihood to adopt an appropriate working position, and less likely to lift weights above 22.7 kg.
Alamgir et al. (2011). **Canada.** 83%	To reduce the risk of patient handling-related musculoskeletal injury	Pre and post intervention evaluation	Healthcare Assistants. *n* = 503	Nursing home	Questionnaire	-Installation of overhead ceiling lifts -A peer coaching and mentoring program	- 64.5% to 80.5% increase in the number of staff who used patient lifts constantly.
Chhokar et al. (2005). **Canada.** 75%	To evaluate the efficacy of overhead lifts in reducing the risk of injury beyond that previously reported for the first-year post-intervention	Pre and post intervention evaluation	Nurses and Healthcare Assistants using 128 ceiling lift points.	Nursing home	Facility records including lost workdays, workers’ compensation claims and other cost linked with patient handling injuries.	Installation of overhead lifts	- $238,166 compensation claims saved yearly. -Significant reduction in lost workdays with p value of 0.024
Tompa et al. (2016) **Canada.** 90%	To evaluate whether a peer-coaching program introduced following a previous patient lift intervention was effective and cost-beneficial.	Pre and post intervention review.	Full time nursing staff in 15 care facilities. *n* = 7562	Nursing Home	Panel data, interviews.	-Installation of overhead lifts. -Coaching program.	-Reduced injury rate of 34% during the intervention and 56% post intervention.
O'Brien et al. (2019). **United States of America**. 88%	To determine if Acceptance and Commitment Therapy may be an effective approach in reducing physical injury, assaults and abuse.	Randomised Control Trial	Nurses and Healthcare Assistants. *n* = 71	Nursing home and assisted living facility	Questionnaire, Qualitative observations	A two-session group-based Acceptance and Commitment Therapy intervention.	-A significant reduction in mental health problems in the intervention group compared to the control group (*p* = 0.0.005) -Major decrease in lost workdays (*P* = 0.11) due to injury.
Larsen et al. (2019) **Denmark.** 83%	To examine the effectiveness of a workplace health literacy intervention on pain intensity, Pain-related inconvenience, and sickness absence.	Randomised controlled trial	Healthcare Assistants. *n* = 509	Nursing homes	Text messages and questionnaires	-Cognitive Behavioural Training on pain prevention and management with communication tools. -Structured dialog between staff and management	−7% reduction in pain intensity. -There was no reduction in absenteeism.
Sedlak et al. (2009) **USA.** 80%	To examine the role of the Clinical Nurse Specialist as it relates to the implementation of a Clinical Nurse Specialist -initiated Safe Movement Programme in reducing healthcare workers’ injuries and related costs.	Pre/post implementation evaluation	Nurses and Healthcare Assistants. *n* = 46	Nursing home	Incident reports, workers’ compensation data and questionnaires.	Clinical Nurse Specialist led Safe movement program and specialized equipment.	−75% reduction in employees’ injuries. −73% monthly reduction in reports of over lifting. −93% reduction in staff compensation insurance.

### 2.3 Search outcome

The initial search generated 334 studies from all the databases, with an additional 23 references identified from reference lists (*n* = 357). All records were exported to endnote X9 and duplicates removed. 138 titles and abstracts were screened for relevance, resulting in exclusion of 94 studies. Full text review of 44 studies resulted in a further 29 papers excluded which did not meet the review's inclusion criteria, with 15 papers finally included in the review. Search records were collated and recorded as shown in Fig. 1.

### 2.4 Quality appraisal

The quality of each of the 15 studies in the review was assessed using the Crowe Critical Appraisal Tool (CCAT), a multi design appraisal tool commonly used in health research as it allows the merits of each study step to be evaluated and scored prior to inclusion in the review (Crowe 2013). This Appraisal Tool consists of the user guide and an appraisal form (Supplemental material 1 and 2) used together to appraise the quality of each study reviewed including reliability and validity. The Crowe Critical Appraisal Tool provides a systematic approach to the appraisal of research, with the tool suggesting eight appraisal categories: preliminaries, introduction, design, sampling, data collection, ethical matters, results and discussion. Each category is allocated a score ranging from 0 to 5 whole points (0=no evidence, 5=highest evidence. See supplemental material 3) with 40 as the highest possible total score. The total score for each study was divided by 40 and multiplied by 100 to determine the percentage value. In this review, all 15 of the studies scored above 72% which was accepted as an indication of sufficient quality.

## 3 Results

Fifteen studies were reviewed with thirteen studies conducted in nursing homes and two studies conducted in high dependency elderly care assisted facilities. Eight of the studies were conducted in the United State America, five in Canada, one in Germany and one in Denmark. Five of the studies had a sample of Healthcare Assistants only, while the remaining ten included both nurses and Healthcare Assistants. No study used a sample of nurses only.

The studies reviewed were of mixed designs. Twelve studies were quantitative studies while three studies (Peterson et al., 2004; Kozak et al., 2017; O'Brien et al., 2019) had a qualitative component. The interventions described to reduce work**-**related musculoskeletal disorders in the reviewed research consisted of a single intervention in four studies, (e.g. ceiling lifts) and double interventions in four studies (e.g. ceiling lifts and coaching programmes). The remaining seven studies reported multidimensional interventions which included more than two interventions (e.g. staff training, protocol on equipment maintenance, resident transfer team, budgeting). Although some of the studies duplicated the same interventions, there was no reported criteria for intervention combination and no indication of the advantage of a particular combination set.

Using Burnard's (2011) framework for content analysis, the findings were presented under the following categories: Specialised equipment, Staff training, Policies and procedures to reduce work**-**related musculoskeletal disorders and Support and follow up which are discussed below.

### 3.1 Specialised equipment

A notable trend among the interventions was the introduction of handling devices which were referred to as specialised equipment, lifting devices, repositioning devices, and handling equipment. It included floor lifts or mechanical lifts, ceiling lifts or overhead lifts, slide boards and fast rising electric beds. Interventions with lifting devices were highlighted in eleven of the fifteen studies and were in three subsets: ceiling lifts introduced during the study (Chhokar et al., 2005; Miller et al., 2006; Alamgir et al., 2008, 2011; Tompa et al., 2016), floor lifts also introduced during the study (Collins et al., 2004; Kurowski et al., 2012, 2017; Gold et al., al.,2017), and floor lifts that pre-existed prior to the introduction of new interventions (Brophy et al., 2001 and Kozak et al., 2017). These different strategies all reported positive outcomes including reduced injury rates, lost workdays, and compensation claims.

A single intervention with the introduction of ceiling lifts reported immediate effect in reducing work**-**related musculoskeletal disorders. This was attributed to ceiling lifts being easy to use, readily available and more comfortable for clients (Chhokar et al., al.,2005 and Alamgir et al., al.,2008) which resulted in yearly claims savings of $238,166 and Can $ 11, 657.21. Also, Miller et al. (2006) introduced ceiling lifts but further compared the use of ceiling lifts against floor lifts among 74 nurses and Healthcare Assistants. Injury rate reductions were noted with the use of ceiling lifts in this study and 75% of the staff expressed a preference for ceiling lifts use as they required less effort to manipulate.

Double interventions were used by Alamgir et al. (2011) and Tompa et al. (2016) who assessed the effectiveness of a coaching program coinciding with the introduction of ceiling lifts. Injury rates reduced by 34% during the intervention with a 56% reduction post-intervention in Tompa et al. (2016) study which involved 15 care facilities with a large sample of 7562 participants. This finding of a 56% reduction post-intervention equated to the prevention of 14 additional lost injury times (Tompa et al., 2016). Alamgir et al. (2011) also reported an increase in the number of staff who used patient lifts constantly from 64.5% to 80.5%. With pre-existing specialised equipment, the impact of safety training was investigated among 46 staff (Sedlak et al., 2009) which reported 75% reduction in injury rates and 73% monthly reduction in reports of over lifting by staff.

In a multidimensional setting, four studies (Collins et al., 2004; Kurowski et al., 2012; Gold et al., 2017, 2017) introduced specialised equipment in combination with staff training and implementation of protocols for equipment use and maintenance. All four studies reported reduction in work**-**related musculoskeletal disorders and injury claims, but Kurowski et al. (2012) also found that a safe resident handling program encouraged lifting more appropriate weights and maintaining proper body posture at work as contributory to reduced work**-**related musculoskeletal disorders.

Pre and post-injury intervention records were widely used to evaluate the outcome of the introduction of specialised equipment (Chhokar et al., 2005; Alamgir et al., 2008; Miller et al., 2006; Collins et al., 2004; Kurowski et al., 2012; Tompa et al., 2016; Alamgir et al., 2011), and outcomes included reduction in lost work days and injury compensation claims. Financial investment in specialised equipment was also profitable, it was revealed that the total savings from compensation claims in five years was three times the cost of specialised equipment and associated interventions (Brophy et al., 2001). Similarly, there was an immediate reduction in compensation claims and indemnity following injury prevention interventions while the total cost of purchasing specialised equipment, staff training and related cost was recovered in less than 3 years post intervention (Collins et al., 2004)

### 3.2 Staff training

The evidence on the effectiveness of staff training in preventing work**-**related musculoskeletal disorders in this review revealed that the training interventions with the most positive outcomes were designed and executed by management of the care facilities (Collins et al., 2004; Kurowski et al., 2012; Tompa et al., 2016, 2017; Alamgir et al., al.,2011; Tompa et al., al.,2016). Conversely, Peterson et al. (2004) involved 35 Healthcare Assistants in the identification of musculoskeletal stressors within the work environment which formed the basis of the training. The study did not find any significant reduction in pain, discomfort or overall physical and mental health among the participants post training. The variation in outcome was also linked to the timing of the training. For example, training of employees on the use of specialized equipment as part of an induction program reduced injury rate, claims and lost workdays but regular updates was more effective in reducing work**-**related musculoskeletal disorders as staff then had a better understanding of the work environment (Collins et al., 2004; Kurowski et al., 2012, 2017).

Training programmes on proper body posture, physical exercise, and reorganization of work environment by staff was also engaged to reduce stressful trunk postures (Kozak et al., al.,2017). The study employed a Computer- assisted Recording and Long-term Analysis of Musculoskeletal Loads measuring system to compare worker's trunk postures, pre and post intervention. The reported outcome included a significant reduction in stressful trunk postures from 2.5% to 1.0%, and maintenance of proper ergonomic measures 6 months’ post-intervention. Gold et al. (2017) also supported training on physical exercises to enhance flexibility and prevent or reduce lower back pain especially in people with history of lower back pain.

The availability of specialised equipment prior to training interventions was prominent and resulted in positive outcomes (Collins et al., 2004; Kurowski et al., 2012; Tompa et al., 2016, 2017 Alamgir et al. (2011). The addition of the coaching program in these studies was found to have encouraged healthcare staff and enhanced their positive perception of lifting devices.

### 3.3 Policies and procedures to reduce work-related musculoskeletal disorders

Adherence to specific policies and procedures that guide mobility interventions in nursing homes reduced work**-**related musculoskeletal disorders (Collins et al., 2004; Kurowski et al., 2012; Gold et al., 2017, 2017). These policies were also introduced on foot of the availability of specialised equipment and focused on their proper usage and maintenance including timely sling laundering and battery charging, which had a substantial effect on the proper use of specialised equipment and further reduced injury rates. A gradual introduction of safe handling policy programmes was carried out in a group of nursing homes over a period of 3 years by a third party, who used a top to bottom approach (Kurowski et al., 2012, 2017) that involved nursing managers, nurses, and Healthcare Assistants as study participants. The policy implemented mandated compulsory use of specialised equipment for handling of residents as required. Injury rates and compensation claims progressively decreased from one-year post intervention. Also, staff were more likely to maintain a good trunk posture and lift weights below 22.7 kg as part of the policy intervention. Conversely, the approach used by Collins et al., 2004 to implement a zero lift policy program was through the charge nurses who ensured that there was no manual lifting of residents. This was achieved through proper assessment of the resident and the use of appropriate transfer method.

Further highlights on the prevention of lower back pain as a type of work**-**related musculoskeletal disorders was made by Brophy et al. (2001) who investigated the association between the incidence of lower back pain and work**-**related musculoskeletal disorders related claims among all Healthcare Assistants. in a 525-bed facility. The policies on mandatory staff training, and monthly equipment maintenance was implemented which resulted in reduced lower back pain and lost workdays culminating in over 54% reduction in work**-**related musculoskeletal disorders related claims.

### 3.4 Support and follow up

To encourage compliance with the use of specialised equipment, adherence to training programmes and policies implementation interventions were initiated as a follow up and to provide support for staff in a multidimensional setting.

One of such was a Clinical Nurse Specialist led safe movement program to support safe movement of clients which involved 46 staff (Sedlak et al., 2009). This was in an attempt to close the divide between scientific evidence on injury prevention and clinical practice. The Clinical Nurse Specialist, being a staff member, was considered to understand clients, staff and the organisational dynamics and use the knowledge to find a suitable way to implement and coordinate evidence-based strategies to reduce the risk of work**-**related musculoskeletal disorders. The intervention also facilitated staff confidence in using specialised equipment and other injury prevention practices. Results demonstrated a significant reduction in injuries and compensation cost associated with work**-**related musculoskeletal disorders.

Another supportive intervention was the use of therapy sessions as a follow up strategy to prevent work**-**related musculoskeletal disorders. Cognitive Behavioural Therapy reduced pain intensity reported by staff in Larsen et al. (2019) study, but the rate of injury-related absenteeism remained significantly high. O'Brien et al. (2019) also examined therapy sessions as a single intervention to reduce musculoskeletal injuries and investigated if Acceptance and Commitment Therapy was effective in reducing physical injury among 71 healthcare staff in long term residential settings. The intervention group had a 2.5 h X_2 therapy sessions focusing on acceptance, psychological flexibility, mindfulness, present moment focus and willingness to experience discomfort. A comparison with the control group that did not undergo the therapy sessions revealed a significant reduction in missed workdays due to injury and mental health symptoms in the intervention group. This study examined Acceptance and Commitment Therapy as a new approach to injury prevention, but it was limited by the inability to measure the intensity and focus of Acceptance and Commitment Therapy required to produce behavioural changes that would reduce musculoskeletal symptoms. The authors concluded that further research was required on the benefits of Acceptance and Commitment Therapy on direct care staff.

Brophy et al. (2001) investigated 3 interventions to support Healthcare Assistants in a 525-bed facility to prevent lower back pain and work**-**related musculoskeletal disorders related claims. First, resident transfer evaluation team was used to assess and categorize the transfer needs of a resident and to determine the appropriate transfer technique to be used by staff. A re-evaluation of the resident was carried out whenever the capabilities of the resident changed. Second, an accident review committee was set up that probed each accident and recommended follow up actions. Some accidents were residents related due to their health conditions, while staff related accidents were due to non-adherence to safe handling policies. Follow up actions were implemented as required including disciplinary action against staff who failed to adhere to recommended handling guidelines. The last was to enlighten budget personnel on the benefit of allocating funds for specialised equipment in view of the cost and implications of lower back pain. Outcomes of the three interventions by Brophy et al. (2001) indicated a significant reduction in the incidence of lower back pain and lost workdays culminating in over 54% reduction in work**-**related musculoskeletal disorders. In addition, $601,300 was projected to be spent on injury compensation in 5 years, compared to $54,800 being spent as a result of the injury prevention interventions.

An open, inclusive workplace culture between management and staff in older person's services significantly reduced pain intensity but did not reduce lost workdays (Larsen et al., 2019). This intervention was to promote an atmosphere that would encourage dialog between workers and the supervisors about challenges in the work environment that may predispose to work**-**related musculoskeletal disorders. Although a significant reduction in pain intensity was reported, it did not result in reduced absenteeism and other measures to promote health in the workplace and improve outcomes were to be considered**.**

## 4 Discussion

The findings of this integrative literature review suggests that there are effective interventions which can be used to prevent and reduce work**-**related musculoskeletal disorders among healthcare workers in nursing homes and long term residential settings. In line with other studies, (Nelson et al., al.,2006; Waters et al., 2012), this review highlights that the baseline intervention in the prevention of work**-**related musculoskeletal disorders is the availability of varied specialised equipment used to facilitate safe handling of older persons with mobility impairment. The widespread usage may reflect an increasing concern by management about the effects of work**-**related musculoskeletal disorders on workload, absenteeism and related cost, and increasing adherence to mandatory health and safety stipulations (Collins et al., 2006). While lifting devices are predominantly used, ceiling lifts appear to have some advantages over floor lifts in terms of time, injury risk and energy spent in transporting floor lifts to the point of use and more so, where the floor is carpeted. Furthermore, the use of floor lifts exceeds recommended standard limits for pushing and pulling in contrast to ceiling lifts and should therefore be discouraged (Waters et al., 2012). However, the choice of specialised equipment for clients should be based on their specific ambulatory needs to ensure client's comfort and effective use by staff. The safe use of handling equipment by healthcare staff for themselves and for their residents is advocated internationally. As well as being a requirement by residential homes regulatory bodies in Ireland (HIQA-IRELAND 2016) and the United Kingdom (DOH-UK 2015), it has been promoted by the World Health organization as an aspect of the Universal Health Coverage advocated to reduce the cost associated with harm and to improve healthcare efficiency (WHO 2019).

The introduction of specialised equipment within a multidimensional approach including training, coaching, injury reviews and maintenance protocols increases the chance of better outcomes in terms of work**-**related musculoskeletal disorders. Although no particular intervention combination pattern was recommended in the review studies, the benefits of multidimensional intervention approaches was unanimous. Therefore, for optimal results, multiple intervention strategies, including the use of specialised equipment, should be driven by a risk assessment of patients and institutional needs (Hignett 2003). If the prevalence of work**-**related musculoskeletal disorders remains high in the presence of these interventions, emphasis should be on the provision of adequate time and supports to ensure the application of proper techniques by staff when using specialised equipment for client care (D'Arcy et al., 2012). Ongoing audit of equipment and techniques might also help to identify areas that require improvement, reduce practice variation, and serve as a quality assurance tool (Poortaghi et al., 2015).

Knowledge and information on safe patient handling is required by staff and should be communicated in a timely manner and revised regularly. Previous reviews (*Clemes et al., 2010**)* Martimo et al., al.,2008; Verbeek et al., al.,2012), reported the persistence of high levels of musculoskeletal debility following staff training on the proper use of specialized equipment alone. The high level was attributed to other factors for example the non-use of lifts, poor work posture, and psychological strain. Subsequently, Clemes et al. (2010) review discourages manual handling training because it focuses on physical handling including lifting, lowering, pushing and pulling of object but encourages multidimensional training interventions in health and safety which would include proper body posture at work, reorganizing the work environment and physical exercises to enhance flexibility. Therefore, this integrative review adds further weight to using a more holistic approach to reduce work**-**related musculoskeletal disorders.

Another factor which may be of significance is who provides the intervention. Trainings designed by management had more positive outcomes in the review. This may suggest attendance and adherence to safety training is better when there is a top down approach to encourage participation and compliance among staff, as employee training tends to be more effective if it is tailored to the preference of most staff (Schmidt 2007). Therefore, it may be beneficial for management to collaborate with staff in planning suitable training programmes as it has the potential to promote involvement in the home and reduce injury rates (D'Arcy et al., al.,2012).

Staff experience appears to significantly influence injury prevention practices by health care workers. Nelson and Baptiste (2006) synthesised the best practices for safe handling and movement of patients in nursing and concluded that healthcare workers at that time tended to rely on personal experiences rather than scientific evidence in choosing interventions to reduce patient handling injuries. Therefore, an effective training intervention should be careful not to dismiss personal experience but integrate it with available evidence and encourage ownership of the training programmes by the care givers.

The adoption of a ‘no manual lift’ policy as a component of safe handling practices contributes to decreased rates of injury in long term care facilities (Li et al., 2004). Similarly, the World Health Organisation (2017) promotes formulating evidence-based policies in mobility care of older adults, to provide security to both staff and client. Such a policy provides specific regulations and guidelines to reduce inappropriate patient handling by healthcare workers. The initial policy on patient lift was the ‘Zero lift’ policy among healthcare staff implemented in 1992 in the United Kingdom (HSE-UK 1992) which resulted in decreased injury rates among staff. Consequently, this policy has been widely advocated in nursing homes (Rockefeller et al., 2000). Healthcare regulatory authorities, as a part of national legislation stipulate the need for guidelines on safe movement and handling of people (Nelson and Baptiste 2006). Implementation of a Zero or no lift policy indicates a joint responsibility between the management and the employee. Adherence to policies to prevent work**-**related musculoskeletal disorders is enhanced by the availability of specialised equipment. Therefore, from a managerial perspective, such a policy should include an administrative guarantee of the availability of appropriate equipment, adequately maintained for use by healthcare staff to reduce the risk of work**-**related musculoskeletal disorders, since some degree of patient handling is vital in providing good nursing care (Edlich et al., 2004). Likewise, staff are required to adhere to zero lift policy, use the available handling equipment and engage with education and training on safe moving and handling. However, the availability of a zero-lift policy will not address all work**-**related musculoskeletal disorders risk, therefore management need to acknowledge other injury risk factors, facilitate their prevention and deal with affected staff in a supportive and non-punitive manner (Nelson et al., 2006).

Onsite consultation with the resident transfer team provides patient specific guidance to staff on safe patient movement, thereby significantly reducing injuries. This finding is consistent with a review by Nelson and Baptiste (2006) which recommends the implementation and use of a lift team in nursing practice. Experimental studies carried out within hospitals indicate that having a lift team policy and a lift team is effective in reducing injury and lost workdays and has subsequently increased nurses’ years of service (Kutash et al., 2009; Hefti et al., 2003). However, the feasibility of having a dedicated lift team available in smaller residential units needs to be considered, given the cost implications. Norris (2018) proposed having a dedicated safe patient handling officer to oversee injury prevention related to patient handling which might be more feasible and economical.

It emerged from this review that an established protocol for the maintenance of handling equipment including timely sling laundering, battery charging, facilitates the proper use of specialised equipment and further reduces injury rates. This is reinforced by Hignett (2003) in a review which strongly recommends maintenance checks on technological devices to eliminate failures and promote efficiency. The protocol should include regular scheduled equipment maintenance and promote reporting of deterioration of the equipment's efficiency. For example, there is a likelihood of the sling being lost during transportation between the nursing home and the laundering agency and therefore home washing or disposable sling use is advised. Regular review of policies to ensure adherence and effectiveness may reduce harm and injury whereas sudden equipment failure will disrupt care and result in negative consequences (Collins et al., 2006).

The act of supporting and mentoring others is already one of the responsibilities of nurses in the clinical setting to enhance learning and professional development (Chung and Kowalski 2012; NMBI 2015). A Clinical Nurse Specialist is believed to have high competence in demonstrating skills to effect changes in practice and the role is gradually gaining clinical acceptance in different specialties (Meehan and Doody 2020). With positive clinical changes resulting from the activities of a Clinical Nurse Specialist in nursing (Werner 2005; White 2012), it could be harnessed to include support in preventing work**-**related musculoskeletal disorders through the services of a Clinical Nurse Specialist. However, it may not be feasible to fund a Clinical Nurse Specialist post to focus solely on safe moving and handling or to add to the workload of an existing Clinical Nurse Specialist. Therefore, a Train-the-Trainer approach could enhance implementation of measures to prevent and reduce work**-**related musculoskeletal disorders. This intervention provides in-depth training to designated nurses in a unit who in turn trains and guides other staff. The lead nurse is considered best placed to find an appropriate means of introducing training and new interventions within a unit due to understanding the teamwork mechanism of the unit. This approach has been cost effective with positive outcomes in other nursing fields (Beaver et al., 2016; Hsiung et al., 2016) and has the potential to be effective in interventions to reduce work**-**related musculoskeletal disorders in nursing homes.

The use of therapy sessions as a strategy to prevent work**-**related musculoskeletal disorders among nursing staff could be effective in reducing pain intensity and treatment outcome in people with non-specific lower back pain (Smeets et al. (2006). However, the use of therapies like Cognitive Behavioural Therapy and Acceptance and Commitment Therapy is evolving and could be considered a psychological support, with possible synergistic effect between physical and psychological interventions (Richardson et al., 2018). Provision of such services may signal concern for staff wellbeing and a more holistic approach from a managerial perspective which may have positive effects.

The prevention of injury reoccurrence is an essential follow up practice (Hammervold et al., 2019). Injury prevention involves risk management and follow up action advocated following the identified risks. This could include observing personnel techniques, disciplinary actions, fixing faulty equipment and review of residents’ care plans to include appropriate handling techniques. National practice guidelines exist for the follow up of injury events, for both staff and residents in healthcare settings (HSE-Ireland 2017). Follow up interventions have generally been effective in nursing (Strahan et al., 2003). As a follow up measure, clinical audit assesses effectiveness of interventions, enhances risk identification (HIQA 2016) and highlights measures to be taken to prevent or reduce risk which will guide future interventions.

An open culture in the nursing home also provides support to staff and help reduce reported work**-**related musculoskeletal disorders. Such a culture necessitates prompt response by management to issues, and keeping staff informed about possible challenges facing the nursing home. This tends to empower staff and create a sense of belonging that facilitates understanding and teamwork and could subsequently reduce pain perception. Suggestions to achieve this include a flexible work environment, supportive leadership and the maintenance of meaningful relationships with staff and residents (McGilton et al., 2014; Zhang et al., 2016).

It is also beneficial to support staff through adequate budget for the implementation and follow up of injury prevention interventions (Brophy et al., 2001). Allocation of funds to such programmes is likely to increase staff confidence and trust in management to protect them from injury and may enhance cooperation in implementing injury prevention practices.

## 5 Limitations

This review, whilst providing valuable information to impact on prevention and reduction of work**-**related musculoskeletal disorders among healthcare workers is limited by some factors. The decision to exclude studies written in languages other than English was based on the potential for miscommunication associated with translation, whereas studies published in other languages could contribute further to knowledge, understanding and prevention of work**-**related musculoskeletal disorders. The reviewed studies were limited to publications between 2000 and 2020, whilst it is acknowledged that research published outside these dates could add valuable knowledge to the prevention of work-related musculoskeletal disorders.

A non-specific quality assessment tool was used to allow for the inclusion of studies with different methodologies. However, the application format of the CCAT quality tool can be subjective (Harrison et al., 2017). Using an integrative review method allowed for the inclusion of various study designs. However, given the experimental nature of most of the studies reviewed, a meta-analysis of the findings would be of benefit. Some of the studies had small samples limiting generalisability (Peterson et al., al.,2004; Kozak et al., al.,2017; Miller et al., al.,2006; Sedlak et al., al.,2009), acknowledged as limitations by the authors. However, the evidence presented consistently indicates interventions that reduces injury rates, compensation claims and lost workdays, although future research with larger number of participants would be beneficial.

## 6 Implications for practice

An injury free workforce is an essential element to ensure quality nursing care especially for clients with mobility needs. This review reveals that a ceiling lift is the intervention of choice to reduce work**-**related musculoskeletal disorders in nursing homes, as it reduces the stress associated with pushing and pulling.

Risk assessment is vital to determine the individual needs of nursing homes and their residents and should be followed with auditing to determine its effect on work**-**related musculoskeletal disorders. Auditing could be led by the management but should be cascaded to all team members to ensure personal ownership of the programmes and to highlight personal responsibility for self-care.

Educational programmes aimed at reducing work**-**related musculoskeletal disorders should incorporate staff experiences to facilitate positive outcomes and should be provided to all new employees and regular updates provided. In addition, an open inclusive work culture in the nursing home should be encouraged as it promotes teamwork and reduces the intensity of pain reported by workers.

Train-the-trainer roles could be used to implement training interventions where staffing resources limit the employment of a Clinical Nurse Specialist or a designated lead to facilitate safe handling practices.

## 7 Suggestions for future research

A meta-analysis on this subject would be beneficial and might provide an in-depth exploration of similar studies to determine the statistical effects of interventions used to prevent or reduce work**-**related musculoskeletal disorders among health care staff.

An experimental study to ascertain the effectiveness of Cognitive Behavioural Therapy on reduction of work**-**related musculoskeletal disorders in nursing homes is recommended. Such research could focus on methods of administration of Cognitive Behavioural Therapy and its usefulness on alleviation of pain associated with work**-**related musculoskeletal disorders. The client perspective on the most effective handling techniques is an area under researched. Such research could assess clients’ satisfaction and sense of security with the intervention used for mobility. In addition, information provided to the client before and during the movement by the health care staff could be examined to determine its impact on client's cooperation with equipment use and movement which may reduce work**-**related musculoskeletal disorders.

## 8 Conclusion

This review synthesized evidence published between 2000 and 2020 on interventions to reduce work-related musculoskeletal disorders among nurses and healthcare assistants in nursing homes.

Single interventions were predominantly the introduction of specialised equipment as well as Acceptance and Commitment Therapy sessions and they were both effective in reducing work**-**related musculoskeletal disorders. The use of ceiling lifts was advocated over floor lifts as the former required less pushing and pulling which further reduced work**-**related musculoskeletal disorders.

Double interventions which involved handling devices and training programmes had positive outcomes. In contrast, the combination of training programmes with Cognitive Behavioural Therapy did not have a significant reduction in lost workdays but were associated with reduced pain intensity. This review does highlight the value of multidimensional approaches (including specialised equipment, training, policies, procedure, support and follow up of staff) to prevent and reduce work**-**related musculoskeletal disorders.

This literature review may guide healthcare managers and staff involved in the procurement of equipment and in the delivery of care to clients with mobility needs. Identifying the most appropriate interventions to prevent or reduce work**-**related musculoskeletal disorders is significant for the wellbeing of all staff, has economic implications in terms of reduced work**-**related musculoskeletal disorders and absenteeism but ultimately positively impacts on the care of mobility impaired clients.

## Declaration of Competing Interest

The authors declare that they have no known competing financial interests or personal relationships that could have appeared to influence the work reported in this paper.

## References

[bib0001] Aghilinejad M., Choobineh A.R., Sadeghi Z., Nouri M.K., Ahmadi A.B. (2012). Prevalence of musculoskeletal disorders among Iranian steel workers. *Iran. Red Crescent Med. J.*.

[bib0002] Alamgir H., Drebit S., Li H.G., Kidd C., Tam H., Fast C. (2011). Peer coaching and mentoring: a new model of educational intervention for safe patient handling in health care. *Am. J. Ind. Med.*.

[bib0003] Alamgir H., Yu S., Fast C., Hennessy S., Kidd C., Yassi A. (2008). Efficiency of overhead ceiling lifts in reducing musculoskeletal injury among carers working in long-term care institutions. *Injury*.

[bib0004] Andersen L.P., Mikkelsen K.L. (2008). Recall of occupational injuries: A comparison of questionnaire and diary data. *Saf. Sci.*.

[bib0005] Beaver C., Gimbert B., Magnan M. (2016). Using train-the-trainer methodology to validate chemotherapy competencies across a large health system. *Oncol. Nurs. Forum*.

[bib0006] Benavides D., Segura S., Ruiz-Cortés A. (2010). Automated analysis of feature models 20 years later: a literature review. *Inf. Syst.*.

[bib0007] Bettany-Saltikov J. (2016).

[bib0008] Brophy M.O.R., Achimore L., Moore-Dawson J. (2001). Reducing incidence of low-back injuries reduces cost. *AIHAJ-Am. Ind. Hyg. Assoc.*.

[bib0009] Burnard P.N., R. (2011).

[bib0010] Carugno M., Pesatori A.C., Ferrario M.M., Ferrari A.L., Silva F.J.d., Martins A.C., Felli V.E.A., Coggon D., Bonzini M (2012). Physical and psychosocial risk factors for musculoskeletal disorders in Brazilian and Italian nurses. *Cad. Saude Publica*.

[bib0011] Chhokar R., Engst C., Miller A., Robinson D., Tate R.B., Yassi A. (2005). The three-year economic benefits of a ceiling lift intervention aimed to reduce healthcare worker injuries. *Appl. Ergon.*.

[bib0012] Chung C.E., Kowalski S. (2012). Job stress, mentoring, psychological empowerment, and job satisfaction among nursing faculty. *J. Nurs. Educ.*.

[bib0013] Clemes S.A., Haslam C.O., Haslam R.A. (2010). What constitutes effective manual handling training? A systematic review. *Occup. Med.*.

[bib0014] Collins J.W., Nelson A., Sublet V. (2006).

[bib0015] Collins J.W., Wolf L., Bell J., Evanoff B. (2004). An evaluation of a "best practices" musculoskeletal injury prevention program in nursing homes'. Injury Prevention (1353-8047).

[bib0016] Crowe M. (2013).

[bib0017] Cunningham C., Flynn T., Blake C. (2006). Low back pain and occupation among Irish health service workers. *Occup. Med.*.

[bib0018] Da Costa B.R., Vieira E.R. (2010). Risk factors for work-related musculoskeletal disorders: a systematic review of recent longitudinal studies. *Am. J. Ind. Med.*.

[bib0019] Davis K.G., Kotowski S.E. (2015). Prevalence of musculoskeletal disorders for nurses in hospitals, long-term care facilities, and home health care: a comprehensive review. *Hum. Factors*.

[bib0020] D'Arcy L.P., Sasai Y., Stearns S.C. (2012). Do assistive devices, training, and workload affect injury incidence? Prevention efforts by nursing homes and back injuries among nursing assistants. *J. Adv. Nurs.*.

[bib0021] Edlich R., Winters K.L., Hudson M.A., Britt L.D., Long Iii W.B. (2004). Prevention of disabling back injuries in nurses by the use of mechanical patient lift systems. *J. Long Term Eff. Med. Implants*.

[bib0022] Ellapen T.J., Narsigan S. (2014). Work related musculoskeletal disorders among nurses: systematic review. *J. Ergon.*.

[bib0023] Gold J.E., Punnett L., Gore R.J. (2017). Predictors of low back pain in nursing home workers after implementation of a safe resident handling programme. *Occup. Environ. Med.*.

[bib0024] Hammervold U.E., Norvoll R., Aas R.W., Sagvaag H. (2019). Post-incident review after restraint in mental health care-a potential for knowledge development, recovery promotion and restraint prevention. A scoping review. *BMC Health Serv. Res.*.

[bib0025] Harcombe H., Herbison G.P., McBride D., Derrett S. (2014). Musculoskeletal disorders among nurses compared with two other occupational groups. *Occup. Med.*.

[bib0026] Harrison J.K., Reid J., Quinn T.J., Shenkin S.D. (2017). Using quality assessment tools to critically appraise ageing research: a guide for clinicians. *Age Ageing*.

[bib0027] Health Information and Quality Authority (2016). https://www.hiqa.ie/sites/default/files/2017-01/National-Standards-for-Older-People.pdf.

[bib0028] Health and Safety Authority (2020). Annual review of workplace injury, illness and fatality statistics 2018-2019. Dublin: Health Saf. Authority.

[bib0029] Hefti K.S., Farnham R.J., Docken L., Bentaas R., Bossman S., Schaefer J. (2003). Back injury prevention: a lift team success story. *AAOHN J.*.

[bib0030] Hignett S. (2003). Intervention strategies to reduce musculoskeletal injuries associated with handling patients: a systematic review. *Occup. Environ. Med.*.

[bib0031] Hsiung Y., Li I., Yeh P.C. (2016). Train the trainer: use of motivational interviewing in end-of-life counselling. *Oncol. Nurs. Forum*.

[bib0032] Nursing and MidwiferyBoard of Ireland, NMBI (2015). https://www.nmbi.ie/Education/Higher-Education-Institutions/Approvals-Midwifery-Programmes/Clinical-Practice.

[bib0033] Health Service Executive (2017). https://www.hse.ie/eng/about/qavd/riskmanagement/risk-management-documentation/hse-integrated-risk-management-policy-part-2-risk-assessment-and-treatment.pdf.

[bib0034] Kozak A., Freitag S., Nienhaus A. (2017). Evaluation of a training program to reduce stressful trunk postures in the nursing professions: a pilot study. *Ann. Work Exposures Health*.

[bib0035] Krippendorff K. (2018).

[bib0036] Kromark K., Dulon M., Beck B.-.B., Nienhaus A. (2009). Back disorders and lumbar load in nursing staff in geriatric care: a comparison of home-based care and nursing homes. *J. Occup. Med. Toxicol.*.

[bib0037] Kurowski A., Boyer J., Fulmer S., Gore R., Punnett L. (2012). Changes in ergonomic exposures of nursing assistants after the introduction of a safe resident handling program in nursing homes. *Int. J. Ind. Ergon.*.

[bib0038] Kurowski A., Gore R., Roberts Y., Kincaid K.R., Punnett L. (2017). Injury rates before and after the implementation of a safe resident handling program in the long-term care sector. *Saf. Sci.*.

[bib0039] Kutash M., Short M., Shea J., Martinez M. (2009). The lift team's importance to a successful safe patient handling program. *JONA: J. Nurs. Administration*.

[bib0040] Larsen A.K., Thygesen L.C., Mortensen O.S., Punnett L., Jørgensen M.B. (2019). The effect of strengthening health literacy in nursing homes on employee pain and consequences of pain‒a stepped-wedge intervention trial'. *Scand. J. Work Environ. Health*.

[bib0041] Lee S.J., Lee J.H., Gershon R.R.M. (2015). Musculoskeletal symptoms in nurses in the early implementation phase of California's safe patient handling legislation. *Res. Nurs. Health*.

[bib0042] Martimo K.-.P., Verbeek J., Karppinen J., Furlan A.D., Takala E.-.P., Kuijer P.P.F.M., Jauhiainen M., Viikari-Juntura E (2008). Effect of training and lifting equipment for preventing back pain in lifting and handling: systematic review. *Br. Med. J.*.

[bib0043] McGilton K.S., Boscart V.M., Brown M., Bowers B. (2014). Making tradeoffs between the reasons to leave and reasons to stay employed in long-term care homes: Perspectives of licensed nursing staff. *Int. J. Nurs. Stud.*.

[bib0044] Meehan M., Doody O. (2020). The Role of the Clinical Nurse Specialist Multiple Sclerosis, the patients’ and families’ and carers’ perspective: an integrative review. *Mult. Scler. Relat. Disord.*.

[bib0045] Miller A., Engst C., Tate R.B., Yassi A. (2006). Evaluation of the effectiveness of portable ceiling lifts in a new long-term care facility. *Appl. Ergon.*.

[bib0046] Moher D., Liberati A., Tetzlaff J., Altman D.G. (2009). Preferred reporting items for systematic reviews and meta-analyses: the PRISMA statement. *Ann. Intern. Med.*.

[bib0047] Nelson A., Baptiste A.S. (2006). Evidence-based practices for safe patient handling and movement. *Clin. Rev. Bone Min. Metab.*.

[bib0048] Nelson A., Matz M., Chen F., Siddharthan K., Lloyd J., Fragala G. (2006). Development and evaluation of a multifaceted ergonomics program to prevent injuries associated with patient handling tasks. *Int. J. Nurs. Stud.*.

[bib0049] Norris K. (2018). International Conference on Applied Human Factors and Ergonomics.

[bib0050] O'Brien W.H., Singh R., Horan K., Moeller M.T., Wasson R., Jex S.M. (2019). Group-based acceptance and commitment therapy for nurses and nurse aides working in long-term care residential settings. *J. Alternat. Complement. Med.*.

[bib0051] Peterson E.L., McGlothlin J.D., Blue C.L. (2004). The development of an ergonomics training program to identify, evaluate, and control musculoskeletal disorders among nursing assistants at a state-run veterans' home. *J. Occup. Environ. Hyg.*.

[bib0052] Poortaghi S., Salsali M., Ebadi A., Rahnavard Z., Maleki F. (2015). Findings from a nursing care audit based on the nursing process: a descriptive study. *Nurs. Midwifery Stud.*.

[bib0053] Punnett L., Wegman D.H. (2004). Work-related musculoskeletal disorders: the epidemiologic evidence and the debate. *J. Electromyogr. Kinesiol.*.

[bib0054] Richardson A., McNoe B., Derrett S., Harcombe H. (2018). Interventions to prevent and reduce the impact of musculoskeletal injuries among nurses: A systematic review. *Int. J. Nurs. Stud.*.

[bib0055] Rockefeller K., Silverstein B., Howard N. (2000).

[bib0056] Russell H., Maître B., Watson D. (2016). Work-related musculoskeletal disorders and stress, anxiety and depression in ireland: evidence from the QNHS'. 2002–2013. *Econ. Soc. Res. Inst. (ESRI) Res. Series*.

[bib0057] Santos C.M.D.C., Pimenta C.A.d.M., Nobre M.R.C. (2007). The PICO strategy for the research question construction and evidence search. *Rev. Lat. Am. Enfermagem*.

[bib0058] Schmidt S.W. (2007). The relationship between satisfaction with workplace training and overall job satisfaction. *Hum. Resour. Dev. Q.*.

[bib0059] Sedlak C.A., Doheny M.O., Jones S.L., Lavelle C. (2009). The clinical nurse specialist as change agent: reducing employee injury and related costs. *Clin. Nurse Spec.*.

[bib0060] Simon M., Tackenberg P., Nienhaus A., Estryn-Behar M., Conway Maurice, Hasselhorn H.M (2008). Back or neck-pain-related disability of nursing staff in hospitals, nursing homes and home care in seven countries—results from the European NEXT-Study. *Int. J. Nurs. Stud.*.

[bib0061] Smeets R.J.E.M., Vlaeyen J.W.S., Kester A.D.M., Knottnerus J.A. (2006). Reduction of pain catastrophizing mediates the outcome of both physical and cognitive-behavioral treatment in chronic low back pain. *J. Pain*.

[bib0062] Smith D., Choi J.-w., Ki M., Kim J.-y., Yamagata Z. (2003). Musculoskeletal disorders among staff in South Korea's largest nursing home. *Environ. Health Prevent. Med.*.

[bib0063] Strahan E., McCormick J., Uprichard E., Nixon S., Lavery G. (2003). Immediate follow-up after ICU discharge: establishment of a service and initial experiences. *Nurs. Crit. Care*.

[bib0064] Tompa E., Dolinschi R., Alamgir H., Sarnocinska-Hart A., Guzman J. (2016). A cost-benefit analysis of peer coaching for overhead lift use in the long-term care sector in Canada. *Occup. Environ. Med.*.

[bib0065] United Kingdom, Health and Safety Executive (1992). https://www.hse.gov.uk/pUbns/priced/l23.pdf.

[bib0066] United Kingdom, Department of Health, Social Services and Public Safety (2015). https://www.rqia.org.uk/RQIA/media/RQIA/Resources/Standards/nursing_homes_standards_-_april_2015.pdf.

[bib0067] United Kingdom, Health and Safety Executive (2019). https://www.hse.gov.uk/statistics/causdis/msd.pdf.

[bib0068] Verbeek J.H., Martimo K.-.P., Kuijer P., Karppinen J., Viikari-Juntura E., Takala E.-.P. (2012). Proper manual handling techniques to prevent low back pain, a Cochrane systematic review. Work.

[bib0069] Wang Y.N., Yan P., Huang A.M., Dai Y.L. (2017). Status quo of injury of nursing personnel with occupational musculoskeletal disorders and their protection knowledge, attitude and behaviour in third grade hospitals. *Chin. Nurs. Res.*.

[bib0070] Waters T.R., Dick R., Lowe B., Werren D., Parsons K. (2012). Ergonomic assessment of floor-based and overhead lifts. *Am. J. Safe Patient Handling Movement*.

[bib0071] Werner H. (2005). The benefits of the dysphagia clinical nurse specialist role. *J. Neurosci. Nurs.*.

[bib0072] White B. (2012). The role of the multiple sclerosis specialist nurse in counselling patients at diagnosis. *Br. J. Neurosci. Nurs.*.

[bib0073] Whittemore R., Knafl K. (2005). The integrative review: updated methodology. *J. Adv. Nurs.*.

[bib0074] World Health Organisation (2017). https://www.who.int/ageing/global-strategy/en/.

[bib0075] World Health Organisation (2018). https://www.who.int/news-room/fact-sheets/detail/ageing-and-health.

[bib0076] World Health Organisation (2019). https://www.who.int/patientsafety/en//.

[bib0077] Zhang Y., Flum M., Kotejoshyer R., Fleishman J., Henning R., Punnett L. (2016). Workplace participatory occupational health/health promotion program: facilitators and barriers observed in three nursing homes. *JoJ Gerontol Nurs*.

